# Biofabrication and Bone Tissue Regeneration: Cell Source, Approaches, and Challenges

**DOI:** 10.3389/fbioe.2017.00017

**Published:** 2017-03-23

**Authors:** Monia Orciani, Milena Fini, Roberto Di Primio, Monica Mattioli-Belmonte

**Affiliations:** ^1^Department of Molecular and Clinical Sciences, Università Politenica delle Marche, Ancona, Italy; ^2^Laboratory of Preclinical and Surgical Studies, Rizzoli Orthopedic Institute, Bologna, Italy

**Keywords:** bone regeneration, cell source, biofabrication, biocompatibility, stem cells and regenerative medicine

## Abstract

The growing occurrence of bone disorders and the increase in aging population have resulted in the need for more effective therapies to meet this request. Bone tissue engineering strategies, by combining biomaterials, cells, and signaling factors, are seen as alternatives to conventional bone grafts for repairing or rebuilding bone defects. Indeed, skeletal tissue engineering has not yet achieved full translation into clinical practice because of several challenges. Bone biofabrication by additive manufacturing techniques may represent a possible solution, with its intrinsic capability for accuracy, reproducibility, and customization of scaffolds as well as cell and signaling molecule delivery. This review examines the existing research in bone biofabrication and the appropriate cells and factors selection for successful bone regeneration as well as limitations affecting these approaches. Challenges that need to be tackled with the highest priority are the obtainment of appropriate vascularized scaffolds with an accurate spatiotemporal biochemical and mechanical stimuli release, in order to improve osseointegration as well as osteogenesis.

## Introduction

Bone is composed of bone tissue and bone marrow encased within the periosteum, a thin strip of soft tissue that envelops the midshafts of long bones, extending to their proximal and distal metaphyses and adjacent epiphyses (Malizos and Papatheodorou, 2005). Bone has the ability to self-repair and regrowth: postnatal bone maintains an intrinsic ability for well-ordered growth, remodeling to satisfy mechanical needs, and renewal after damages.

In large bone defects (caused by significant trauma or systemic disease, pathological fractures, non-union, infections or compromised blood supply) this capability can however fail, resulting in permanent defects that can lead to a loss of function. Bone regenerative ability declines with age; therefore, there is a need for *ad hoc* treatments in patients with skeletal diseases determined by the rise in population aging. It must be also stressed that the next most transplanted tissue after blood is bone (Leach and Mooney, 2004; Oryan et al., 2014).

In bone defect treatments, the “gold standard” remains bone grafting (Brydone et al., 2010): bone graft could be used alone or in combination with other materials in order to promote bone healing through osteoinduction, osteoconduction, and osteogenesis. An ideal bone graft can be in the form of autograft (harvested from the patient), allograft, or xenograft (obtained from a donor or animal), or represented by the use of an engineered synthetic biomaterial (Gibbs et al., 2014). Table 1 summarized the advantages and disadvantages of possible different bone grafts (Tang et al., 2016).

**Table 1:** **Advantages and disadvantages of different bone grafts**.^a^

	Advantages	Disadvantages
Autograft	Osteogenic Osteoconductive Osteoinductive	High patient morbidity: pain and infection at donor site, possible visceral injury during harvesting Lack of vascularization Limited availability and quantity
Allograft or xenograft	Osteoconductive Osteoinductive High availability No donor site morbidity	Lack of osteogenicity and vascularization Relatively higher rejection risk Risk of disease transmission High cost
Engineered grafts	Capability to integrate growth factors and stem cells for osteogenicity and graft incorporation improvement Shaped to fit site defects No donor site morbidity	Osteogenicity limited by material porosity (due to manufacturing process) Variable biodegradability of different materials Poor neovascularization Unknown immune response Limited mechanical properties

*^a^Modified from Gibbs et al. (2014) and Tang et al. (2016)*.

The intrinsic reparative capacity of bone grafts represent the natural model to reproduce when using new therapeutic options in tissue engineering strategies: appropriate scaffolds, growth factors, and/or cells, has, in some cases, improved grafts incorporation, osteoconductivity, osteoinductivity, and osseointegration (Kundu et al., 2014). Scaffolds must support cell colonization, proliferation, differentiation, and migration. They usually entail a solid load carrying structure with an intersected pore network, whereas hydrogels containing encapsulated cells often form the “matrix” (Bose et al., 2012). Scaffolds should possess appropriate physicochemical properties (i.e., stiffness, biodegradability, surface chemistry, etc.) that are essential for tissue formation and be capable to face mechanical stresses (Table 2).

**Table 2:** **Scaffold features for bone tissue engineering strategies**.^a^

Biocompatibility	Capability to support normal cell activity with no toxic effect in host tissues particularly during degradation
	Osteoconductive, osteoinductive, and osteogenic properties
	Ability to promote angiogenesis for new blood vessels formation around the implant are advisable
Biodegradability (bioresorbability)	Controlled degradation of a scaffold with time is mandatory to generate space for the growth of new bone tissue and, eventually, the replacement of the synthetic scaffold
	Degradation rate of can be tailored to the application required (e.g., controlled release of biomolecules)
Pore size and porosity	Critical feature for the diffusion of oxygen and nutrients for cell survival and proliferation
	Minimum pore size of 100 μm
	Pore sizes of 200–350 μm are optimal for bone tissue ingrowth
	Meso-porous structures (micro- and macro-porosities mixture) are better than macro-porous ones in supporting cell adhesion
	Porosity influences scaffold’s mechanical strength
Mechanical properties	Should be in line with host bone properties in facing mechanical stress and reacting to load transfers
	Differences in the topography and mechanical characteristics between cortical and trabecular bone affect scaffold design

*^a^Modified from Tang et al. (2016) and Bose et al. (2012)*.

## Cell Source

Cells are commonly used to repair injured tissue, as they are physiologically involved in tissue development and homeostasis. Osteoblast and osteocyte are the key regulators of bone deposition, modeling, and remodeling. Therefore, osteoblasts and/or their precursors represent an excellent cell source for a successful cell-based skeletal treatment.

The incorporation of mesenchymal stromal cells (MSCs) into bone tissue engineering strategies has been a crucial progress. The most frequently used are bone marrow stromal cells (BM-MSCs) (Oreffo et al., 2005; Robey, 2011; Dawson et al., 2014), given to the fact that they have been broadly studied, but several different sources have also been exploited (Table 3).

**Table 3:** **Mesenchymal stromal cells (MSCs) in bone tissue engineering**.^a^

	Potential for bone tissue engineering	Advantages	Disadvantages
Bone marrow	Osteogenic Potential for neovascularization	Relatively easy acquisition *In situ* recruitment Well-characterized	Donor morbidity Limited proliferative potential Fewer cells compared to other sources Cell number Related to age and health of donor
Adipose tissue	Osteogenic Potential for neovascularization	Easy acquisition Well-characterized	Donor morbidity (due to anesthesia)
Oral cavity MSCs (dental pulp, periodontal ligament)	Osteogenic	Abundant Easy acquisition	Not well-characterized
Skin	Potential for neovascularization Support to osteogenic differentiation	Abundant Minimal donor morbidity	Not well-characterized
Periosteum	Osteogenic	Well-characterized *In situ* recruitment Can be co-seeded with bone marrow-derived stem cells	Cell number and activity related to donor age

*^a^Modified from Tang et al. (2016)*.

Viable alternatives to BM-MSCs are represented by adipose tissue-derived stem cells (ASCs) and oral cavity MSCs. ASCs have a documented *in vitro* osteogenic aptitude (El Tamer and Reis, 2009), ease of access, and abundance (Szpalski et al., 2012) and, moreover, survive in low oxygen and/or glucose environments. The latter aspects are an intriguing advantage when the blood and nutrient supply are limited, like with biofabricated bone constructs. Oral cavity MSCs have been exploited for bone tissue engineering strategies (Orciani et al., 2012; Liu et al., 2015a,b) and also proposed for biofabrication of bone (El Tamer and Reis, 2009; Wang et al., 2011; Zhu and Liang, 2015).

Another attractive stem cell reservoir that could meet bone tissue engineering criteria is the skin basal layer (Takeda et al., 1992; Orciani and Di Primio, 2013). The so-called skin-derived multipotent stromal cells, isolated from this site, show multipotent differentiation ability, and are capable to become adipocytes, osteoblasts, chondrocytes, neurons, and pancreatic cells (Orciani et al., 2010). These features in combination with their immunosuppressive effect make them an ideal challenger for various cell transplantation therapies (Vishnubalaji et al., 2012).

The growth, development, and regeneration of bone as well as cartilage rest on periosteum presence. This tissue is pluripotent (Arnsdorf et al., 2009) and may be used for engineering *in vivo* new bone formation (Castro-Silva et al., 2012; Ferretti and Mattioli-Belmonte, 2014). Osteoblasts and chondroblasts of periosteum are well characterized in terms of function, gene expression, cell and extracellular protein synthesis, and secretion as well as structural organization for the elaboration of bone and cartilage, respectively (Colnot, 2011; Mafi et al., 2011). Fewer investigations are relative to the nature and role of periosteum in the bone and cartilage formative and repair processes, or to possible differences and effects of various periosteum sources on osteogenesis and chondrogenesis. As far as animal is concerned, periosteum from only certain bones and at different ages was examined (O’Driscoll and Fitzsimmons, 2001; O’Driscoll et al., 2001; Fan et al., 2008), and results of these and related studies (Kwon et al., 2002; Szulc et al., 2006) could not be easily correlated. Moreover, the analysis of tissues from different anatomical regions of single calves showed that each periosteum retained its own gene expression, protein and proteoglycan secretion, growth, and development (Kusuhara et al., 2009). In 2011, Matsushima and co-workers compared the osteogenic and chondrogenic potential of periosteal tissue harvested from individual young calve sites undergoing intramembranous (cranium and mandible) or endochondral ossification (radius and ilium) by implantation as tissue-engineered constructs in nude mice. They demonstrated that the osteogenic and chondrogenic ability of the different constructs depended on the periosteal source, regardless of intramembranous or endochondral ossification, as cranial and mandibular periosteal tissues were able to enhance bone formation most and least conspicuously, respectively (Matsushima et al., 2011).

In humans, recent researches have been devoted to the evaluation of periosteal cells differentiation ability in response to mechanical, chemical (e.g., growth factors) stimuli as well as in cocultures (Ferretti et al., 2014; Mattei et al., 2015; Dicarlo et al., 2016). Moreover, researches related to possible changes associated with donor age showed that this parameter affects periosteal-derived stem cell behavior mainly in term of bone remodeling (Ferretti et al., 2014).

The influence of stem cells has been tested in animal models; however, it is still unclear whether these cells do retain the *in vivo* capacity to form bone (Marolt et al., 2010). At last, in order to meet the good manufacturing practice standards, a well-defined, standardized protocol for the isolation and *in vitro* manipulation of these cells is still necessary (Seong et al., 2010; Tare et al., 2012).

**Figure 1:**
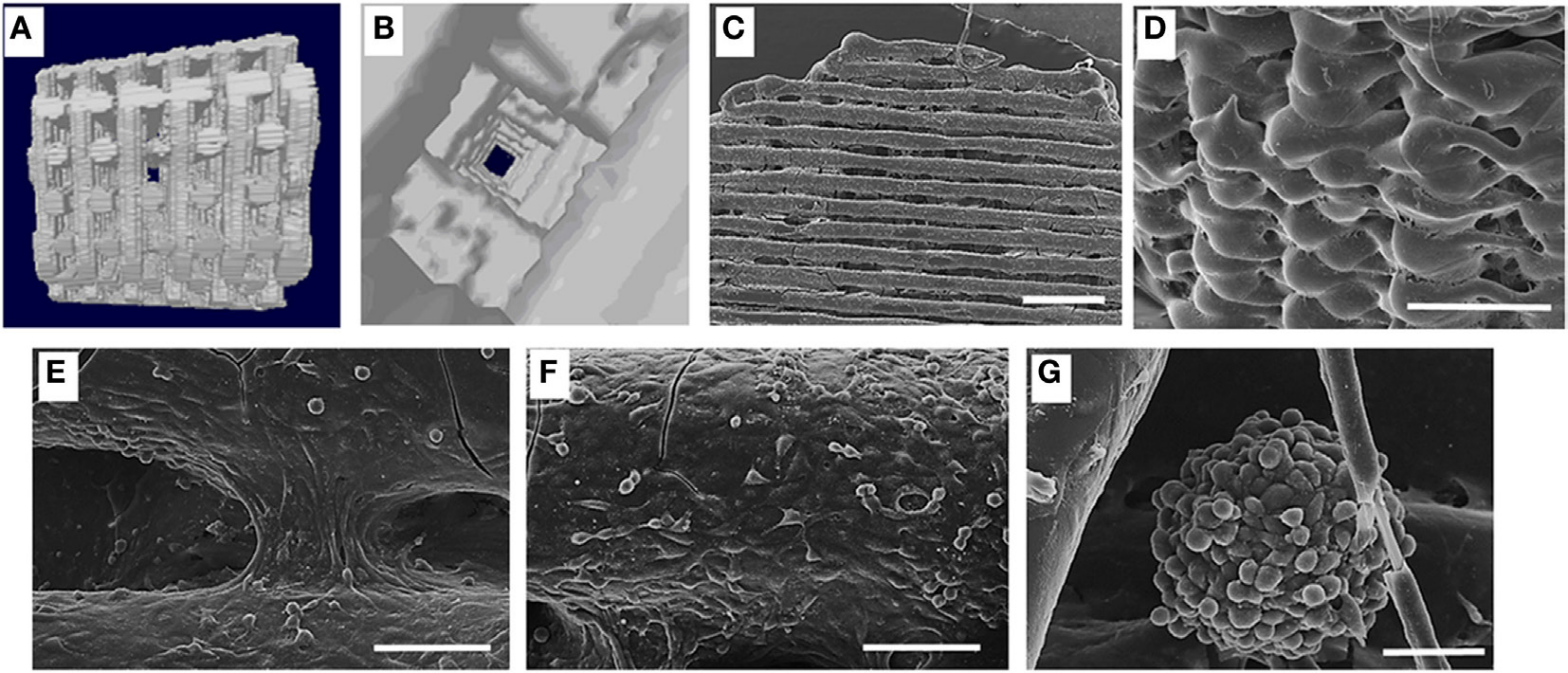
**(A)** Lateral view of three-dimensional (3D) fiber deposited polycaprolactone (PCL) scaffold reconstruction; **(B)** inners structure of the same scaffold; **(C)** colonization of the 3D PCL scaffold in dynamic condition; scale bar 1 mm; **(D)** high magnification showing the external cell monolayer, scale bar 1 mm; **(E)** cells bridging the grooves, scale bar 100 μm; **(F)** cell arrangement suggestive of a new vascular structure, scale bar 100 μm; **(G)** spheroid of MC63 and human umbilical vein cells generated in dynamic condition scale bar 50 μm [from Kyriakidou et al. (2008)].

Angiogenesis is critical in creating a viable biofabricated bone construct, for this reason, the use of two, rather than one, cell types has gathered much interest in bone tissue engineering strategies (Kyriakidou et al., 2008) (Figure 1). For instance, BM-MSCs were used to generate blood vessels also seeded onto a scaffold with endothelial cells (Fedorovich et al., 2010; Gao et al., 2014). Indeed, the inefficient stimulus for a rapid development of new blood vessels that invade the coculture grafts may explain the fail in proving bone formation using this method (Liu et al., 2015a,b; Unger et al., 2015), making this approach still limited.

## Biochemical Signaling for Bone Biofabrication

Bone tissue engineering has tried to exploit the regenerative properties of bone physiological processes (Table 4). *In vivo*, biochemical signals (i.e., growth factors, hormones, and cytokines), secreted locally in the areas undergoing bone remodeling or at the injury sites, cause the migration of inflammatory and precursor cells and/or the activation of osteoblasts and osteoclasts (Kanczler and Oreffo, 2008). Activation of bone-forming and resorbing cells determine to new bone generation during the healing or the remodeling process, respectively. Clinical studies usually utilize growth factors at the range of milligrams per milliliter instead of nanograms per milliliter: this results in adverse effects such as ectopic bone formation, antibody development, and, as latter event, carcinogenesis. Indeed, conflicting data on the appropriate doses of growth factors for bone tissue engineering strategies are available, with a broad range of concentrations in use (Gothard et al., 2014). Moreover, it is still unclear (i) which dose of growth factors is actually delivered *in vivo* by the constructs within a bone defect (Santo et al., 2013) and (ii) the effects of the co-use of multiple growth factors (Young et al., 2009; Kuhn et al., 2013).

**Table 4:** **Growth factors and bone tissue engineering**.^a^

Growth factor	Tissues	Effects
Bone morphogenetic protein (BMP) 2 and 7	Bone, cartilage	Osteoblast differentiation and migration
		Accelerated bone healing
Fibroblast growth factors 1, 2, and 18	Bone, muscle, blood vessel	Endothelial cell migration, proliferation, and survival
		Increased osteogenic differentiation of mesenchymal stromal cells
Insulin-like growth factor-1	Bone, cartilage, muscle	Osteoprogenitor cell proliferation and differentiation
Platelet-derived growth factor (PDGF)-AA and PDGF-BB	Bone, cartilage, blood vessel, muscle	Endothelial cell proliferation, migration, and growth
		Osteoblast replication *in vitro*
		Type 1 collagen synthesis
Parathyroid hormone and parathyroid hormone-related protein	Bone	Intermittent dosage → stimulation of osteoblasts → increased bone formation
		Continuous administration → bone resorption
Transforming growth factor-β3	Bone, cartilage	Bone-forming cell proliferation and differentiation
		Enhancement of *in vivo* hyaline cartilage formation
		Antiproliferative effect on epithelial cells
Vascular endothelial growth factor	Bone, blood vessel	Enhancement of vasculogenesis and angiogenesis (functionality of vasculature is concentration dependent)
		Reduction or increase in bone formation dependent on concentration when used in combinational with BMP-2 delivery

*^a^Adapted from Tang et al. (2016) and Gothard et al. (2014)*.

Delivery of biochemical cues can be obtained in different ways: unbound, bound within the implant for a controlled delivery, coated on the implant surface, or coded within the cells *via* gene delivery mechanisms (Zhang et al., 2010; Catros et al., 2012). In the first case, we observe a burst release of growth factors, resulting in a rapid clearance from the microenvironment: this technique may be appropriate for an immediate stimulus and it is strictly dependent on the biomaterial degradation rate. Physical entrapment or covalent binding is a more appropriate approach when a prolonged, more controlled, or on-demand release of the growth factor or drug is required (Zhang et al., 2010; Mourino et al., 2013). With these techniques, a sustained release over a 15-day period was demonstrated for active lysozyme enclosed within thermoresponsive PLGA microspheres incorporated into extrusion printed PEG-PLGA constructs (Sawkins et al., 2015).

Three-dimensional (3D) printing methods can create bioactive or bioinstructive scaffolds, incorporating growth factors or drugs with a spatiotemporal distribution. For instance, the spatial patterns of bone morphogenetic protein (BMP)-2, generated on a fibrin surface using an inkjet bioprinter, was able to differently affect murine muscle-derived stem cells: cells seeded onto the BMP-2 pattern undergo osteogenic differentiation as evidenced by alkaline phosphatase activity, while those seeded outside the BMP-2 pattern remain undifferentiated (Phillippi et al., 2008). A reduction in biological activity of bioprinted recombinant human BMP-2 was also observed (Vorndran et al., 2010). Since the majority of growth factors have an *in vivo* short half-life, it is important to take into consideration both the biochemical molecule properties and scaffold features.

Currently, in tissue engineering, hydrogels are the most investigated polymers for cell encapsulation and biochemical cues *in situ* delivery. Molecules such as BMP-2 and transforming growth factor-β3 were incorporated into alginate hydrogels designed to degrade at different rates by gamma-irradiation, and the effect of single and dual growth factors delivery on encapsulated rat BM-MSCs was investigated. The appropriate controls of scaffold degradation rate made possible the modulation of osteogenesis (Simmons et al., 2004). In order to mimic the function performed by the extracellular matrix (ECM), bioactive hydrogels containing protease sensitive sites, cell adhesion molecules such as RGD-containing peptides, and/or biological cues in the form of growth factors, inorganic minerals, or drugs, were also developed (He et al., 2008; Fedorovich et al., 2011).

**Figure 2:**
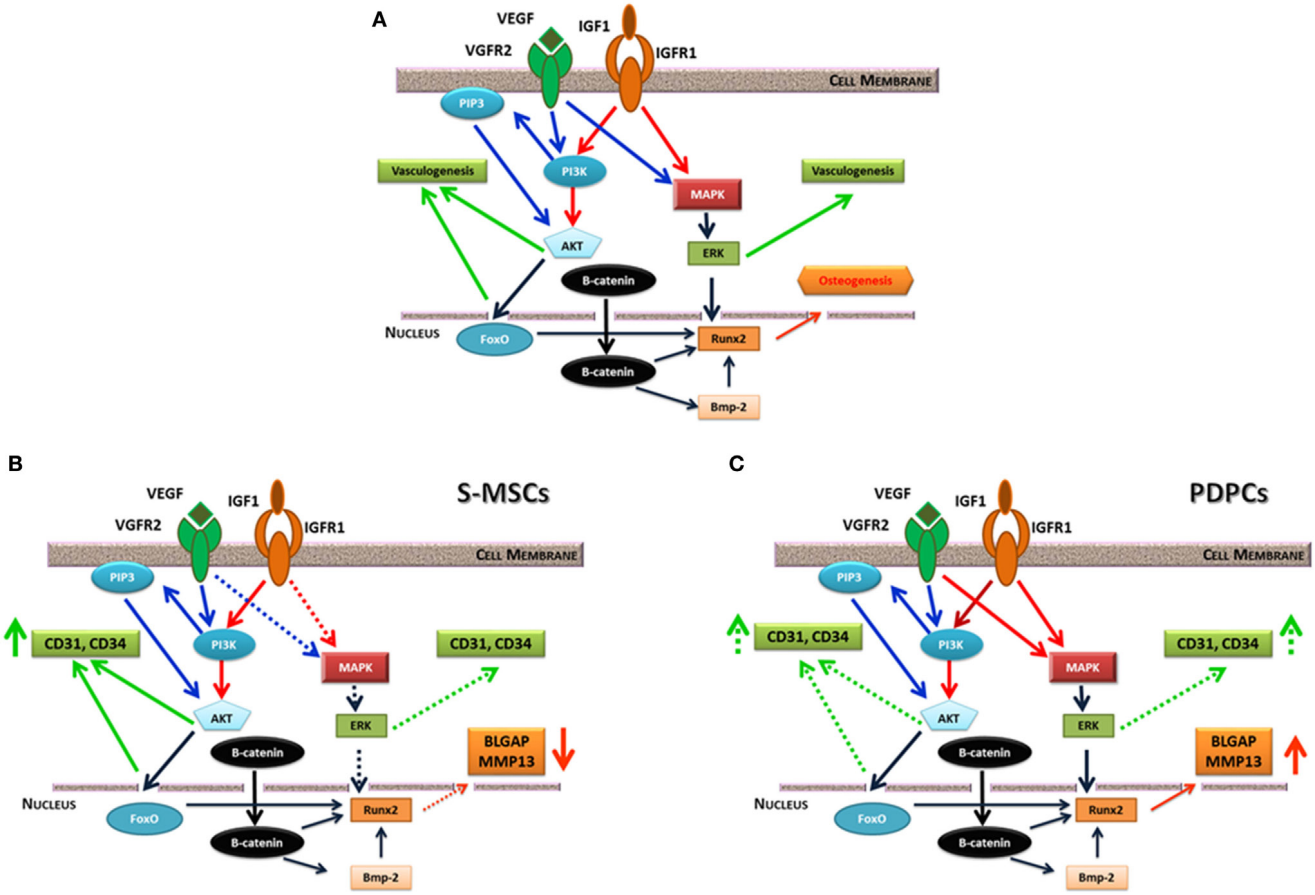
**(A)** Possible interplay between insulin-like growth factor (IGF)-1 and vascular endothelial growth factor (VEGF) receptor signaling pathways. They equally converge on ERK kinase, which fine-tune the activity of the main osteogenic transcription factor Runx2. IGF1 and VEGF also trigger PI3/AKT signaling pathway that can induce vasculogenesis or beta-catenin phosphorylation. On the contrary, non-phosphorylated beta-catenin acts as a pro-osteogenic factor regulating Runx2 and other genes. **(B)** consequences of IGF1 or IGF1/VEGF treatment on skin-derived multipotent stromal cells (S-MSCs) gene expression suggesting their commitment toward the endothelial phenotype. **(C)** Consequences of IGF1 or IGF1/VEGF treatment on PDPCs showing their main commitment toward the osteoblastic phenotype. Continuous lines indicate a marked effect, while dotted lines suggest a weaker regulation. Modified from Ferretti et al. (2014).

Vascular endothelial growth factor (VEGF) plays a key role in angiogenesis during bone development. Several studies investigated the consequence of VEGF, or of a combination of growth factors, on angiogenesis in engineered bone constructs. The co-immobilization of VEGF and angiopoietin-1 on 3D porous collagen scaffolds increased endothelial cell proliferation *in vitro* and in organotypic cultures (Chiu and Radisic, 2010). Studies on synergistic or cumulative effect of VEGF and of insulin-like growth factor underlined as these molecules elicit different cell response in term of bone formation and angiogenesis in relation to the stem cell origin (Ferretti et al., 2014; Dicarlo et al., 2016). Moreover, this different commitment is linked to a diverse mitogen-activated protein kinase or PI3K/AKT signaling pathway activation (Figure 2) [see Ferretti et al. (2014) and Dicarlo et al. (2016) for details].

## Additive Manufacturing Technologies (AMTs), Biofabrication, and Bone Tissue Engineering Strategies

Rapid prototyping or solid freeform fabrication (i.e., AMT) was developed in the mid-1980s, providing a high level of control of the construct architecture, flexibility to scale-up fabrication, and assuring reproducibility and standardization of the manufacturing process. Scaffolds developed using conventional fabrication techniques lacks in precision and reproducibility; on the contrary, AMT provides customized scaffolds with precise geometries for replacing damaged or diseased tissues and organs. AMT enables the production of 3D artificial implants using many biomaterials able to meet rigorous performance criteria for clinical use (Hutmacher and Cool, 2007) as indicated in the growing use of 3D printed artificial implants instead of traditional metallic ones in hip replacement surgery. In recent years, the increasing attention in generating a high personalized and cost-effective medical therapy not only resulted in the growing use of AMT in the manufacture of 3D tissue-engineered structures (Melchels et al., 2012) but has also determined improvement within AMT techniques.

Usually, AMT allows the setup of 3D objects by means of data generated by computer-assisted design (CAD) software or imported from clinical 3D scanners such as X-ray computed tomography, magnetic resonance imaging, and single-photon gamma rays (SPECT). The CAD model is then transformed to a Standard Tessellation Language (STL) file that guides the 3D printer computer system to generate layer-by-layer the object. The manufacturing of a variety of biomaterials fitting the different AMTs allowed the development of scaffolds with tunable properties (Melchels et al., 2012). The American Society for Testing and Materials International Committee F42 on AMT has divided these technologies into seven different processes, which are in line with the layer deposition and bonding techniques [see Gibbs et al. (2014) and Tang et al. (2016) for details].

At present, cell-based treatments are not yet available for clinical use despite the advances in biofabrication and they rely on manual cell seeding and culturing of pre-fabricated scaffolds (Ferris et al., 2013). The current cell-based therapies are only applicable on a limited scale, since they are operator dependent, time consuming, and often inefficient. Moreover, cell seeding on pre-fabricated scaffolds does not recreate the cell organization of native tissues also in term of vascularization. To address these issues and improve cell spatial distribution within the scaffold, cells could be incorporated by an AMT termed as biofabrication, in order to create living cell/biomaterial/biomolecule constructs.

In bone tissue engineering, biofabrication techniques could provide a means to control uniformity of cell distribution or localization on the scaffold surface (Guillotin et al., 2010; Fedorovich et al., 2012). Moreover, the integration of growth factors within the cellular matrix and/or their incorporation inside the scaffold itself during the printing process provides a method for a controlled drug delivery and release (Khatiwala et al., 2012; O’Brien et al., 2014; Tang et al., 2016).

In bone, cells are at different stages of proliferation, differentiation, and maturation inside multi-layered organized ECM. By biofabrication, it is possible to bioprint cells onto a suitable scaffold to create bone with the ability to maintain cell functional capability as well as allowing bone remodeling (Fedorovich et al., 2011).

Biofabrication could provide a more cost-effective manner for the treatment of patients with musculoskeletal defects or disease in addition to offer a new therapeutic option for patients who cannot be cured with traditional therapy. The possibility to seed cells and biomolecules in a 3D space, with an improving better degree of detail and in a user-controlled, predefined way is a key biofabrication breakthrough over traditional approaches (Table 5). Indeed, accurate printing supports the manufacturing of a customized 3D structure that closely fit the defect, thereby decreasing engraftment chances or injury misrepair. Finally, biofabricated bone will remove the donor bone graft requirement, thereby permitting to the patients to undergo surgery earlier (thus reducing waiting list times while recovering mobility earlier), and with a reduced risk of physical and psychological morbidity. The chance of rejection of a biofabricated bone tissue is further reduced by the use of autologous cells.

**Table 5:** **Applications, advantages, and limitations of printing stem cells and biomolecules**.^a^

	Cells	Biomolecules
Applications	Stem cell genomics Patches for wound healing *Ex vivo* generation of tissue replacement	Protein and DNA arrays Tissue engineering uses
Advantages^b^	Programmable Low cost Three-dimensional complexity High throughput	Programmable Low cost Non-contact, reducing risk of cross-contamination from surface No modification required for proteins or substrates
Disadvantages	Cytocompatibility in both solid and liquid forms Viscosity has to be lower than a threshold as defined by the printing method	Lower resolution compared to state-of-the-art protein array Number of available binding sites on the receiving substrate Cytocompatibility Viscosity

*^a^Modified from Tang et al. (2016)*.

*^b^Compared to conventional methods*.

Several AMTs have been experienced to manufacture 3D scaffolds, and more recently, in the printing of tunable hydrogels for bone tissue engineering. Table 6 summarized some of the AMTs used in bone tissue engineering.

**Table 6:** **Classification and applications of additive manufacturing technology (AMT)**.^a^

AMT	Advantages	Disadvantages	Applications
Stereolithography Two-photon polymerization	High-dimensional accuracy Transparent materials	Single composition Cytotoxic photo-initiator Photopolymer materials only Post-processing mandatory Limited cell printing ability Heterogeneous cell distribution	Clinical implants Surgical guides Tissue engineering scaffolds Cell-incorporated three-dimensional (3D) constructs 3D microvasculature networks
Drop on-demand inkjet printing Poly-jet technology	Fast Wide range of biomaterials Inexpensive existing technology Fabrication of composite structures Multi-cell printing	Nozzle blockage common Low bioink viscosity limits improvement of 3D constructs Poor mechanical strength of 3D constructs	Clinical implants Surgical guides Tissue engineering scaffolds Cell-incorporated 3D constructs Biofabrication
*Non-melting extrusion* 3D bio plotting Solvent-based extrusion free-forming Robocasting Direct-write assembly Electrospinning Pressure-activated microsyringe *Melting extrusion* Fused deposition modeling 3D fiber deposition Multiphase jet solidification	Cheap mechanism with relatively good throughput No post-processing needed Low material waste Cytocompatible Rapid Non-toxic materials with good properties	Low accuracy Poor mechanical strength Precise control of ink rheology necessary Use of solvents Low accuracy Weak bonding between dissimilar polymer layers	Tissue engineering scaffolds Cell-incorporated 3D constructs Biofabrication Clinical implants Tissue engineering scaffolds
Selective laser sintering Selective laser melting Electron beam melting Selective mask sintering	Wide range of biomaterials High material strength Good material properties	Thermal stress and degradation Accuracy limited by particle size Atmosphere control needed for metal printing	Surgical implants with complex structure Tissue-engineered scaffold Medical devices
Laser engineering net shape Laser cladding Directed metal deposition	Wide range of biomaterials Good material properties	Low accuracy Thermal stress Atmosphere control needed for machining process	Orthopedic implants
Laminated object manufacturing Ultrasonic consolidation	Low temperature process	Shrinkage Significant waste Delamination	Orthopedic implants
3D printing	Low temperature process Fast Fabrication of composite structures	Powders are necessary Powder entrapment High porosity Low surface quality Accuracy restricted by particle size Cell-changing environment	Clinical implants Tissue engineering scaffolds

*^a^Modified from Tang et al. (2016)*.

In microextrusion deposition method, thin thermoplastic grains or filaments are wormed up until melting and then piloted by a controlled robotic device, to generate the 3D construct. The fused material is extruded and then it hardens immediately. A temperature just below the solidification point of the material must be maintained in order to guarantee the good interlayer adhesion (Melchels et al., 2012). Fedorovich et al. (2008) demonstrated the possibility to generate with this procedure bone grafts by depositing 3D fibers composed of various hydrogels and goat BM-MSCs with no damage to cells in term of osteogenic differentiation during the printing process. In another study, they developed heterogeneous hydrogel constructs with endothelial progenitor cells and goat multipotent stromal cells to promote neovascularization during bone regeneration (Fedorovich et al., 2010).

Pressure-activated microsyringe (PAM) fabrication is a peculiar microextrusion technique in which the polymer is distributed through a tool-head installed on an arm or on the *z*-axis of a computer-controlled 3D micropositioner. The achieved scaffold resolution is normally a function of the polymeric system viscosity, the motor speed, the physical principle that permits polymer distribution, and the nozzle geometry (Vozzi et al., 2002; Tirella et al., 2012). This technique has been used to modulate different cell cytotype behavior in response of topological features (Mattioli-Belmonte et al., 2008) and, more recently, to generate bioactive glass–poly(lactic-co-glycolic acid) (PLGA) scaffolds mirroring the topological characteristics of cancellous bone (Mattioli-Belmonte et al., 2015).

Laser-assisted bioprinting involve a pulsed laser source, a receiver substrate for patterning and collecting cells and biomaterials, and a target. An essential element is a laser-absorbing interlayer with a high heat transfer coefficient. Individual cells in suspension are “driven” by directed laser beams and deposited onto a solid surface. This cell-by-cell deposition enables a precise cell micropatterning and improves cell interactions (Melchels et al., 2012; Guillotin et al., 2014). This technique was used to bioprint human osteosarcoma cells (MG63) onto a bio-polymeric matrix (Barron et al., 2005) and to create an on-demand pattern of nano-hydroxyapatite and human osteoprogenitor cells (Catros et al., 2011). The majority of cells survived throughout the printing process (Catros et al., 2012) and the layer-by-layer assembly method exceeded the seeding a single locus of the scaffold during the creation of a 3D construct (Tang et al., 2016).

At last, the inkjet-based cell printing is a useful, simple, and low-cost method providing microenvironmental cues to cells in order to increase cell survival or manipulate their morphofunctional behavior. This technique is able to generate microscale organization of deposited cells (Cui and Boland, 2009) without compromising their viability or inducing damage to cell phenotype or genotype (Gao et al., 2014). Inkjet-based cell printing could be used to fabricate complex multicellular constructs, since it allows the simultaneous printing of multiple cell types together with biomolecules alongside biomaterials. Inkjet-based printing is one of the methods used to make cell-laden hydrogels (Ferris et al., 2013). With this technology, Gao et al. (2014) demonstrated that encasing human MSCs in poly(ethylene glycol) dimethylacrylate (PEGDMA), containing either bioactive glass or hydroxyapatite nanoparticles, cells were viable post-printing and a greater osteogenesis was present in the construct containing hydroxyapatite.

## Selecting a Biomaterial

Scaffolds generated with AMT are generally made of ceramic, metal, self-assembly peptides, and synthetic or natural polymers (Stevens et al., 2008; Leijten et al., 2015). Due to the specific advantages and disadvantages of each type of biomaterial, the use of composite scaffolds is becoming more common. Several reviews have comprehensively addressed the most common combinations biomaterials as well as their *in vitro* or *in vivo* investigation for their potential use for bone tissue engineering strategies (Leach and Mooney, 2004; Stevens et al., 2008). Indeed, there is no agreement on which biomaterial (or possible mixture) is optimal for bone biofabrication, and the selection is constrained to the AMT employed. Moreover, some AMTs (e.g., stereolithography) require cytotoxic post-processing procedures, while laser sintering can cause biomaterial thermos degradation, with a loss of minute microstructure that, as a consequence, affects material porosity and cell viability (Stevens et al., 2008; Ferris et al., 2013; Gibbs et al., 2014; Tang et al., 2016).

The increasing advances in materials science and engineering has improved the development of the so-called smart materials, in particular polymeric smart materials, for a wide number of applications including bone tissue engineering ones (Stuart et al., 2010; Kumari et al., 2011; Ribeiro et al., 2015). The smart materials display reproducible, significant, and stable variations of at least one of their property when subjected to external stimuli and are usually classified based on the output response (e.g., piezoelectric materials, shape memory materials, temperature responsive polymers, conductive polymers, etc.) (Jeong and Gutowska, 2002).

The interest in the application of active materials is related to the fact that electrical signals control many of the major function in human cells and organs (Moore, 1975; Foulds and Barker, 1983; Ribeiro et al., 2015). For instance, bone tissue adaptation and remodeling are determined by a feedback mechanism that involves electromechanical processes due to its piezoelectric nature. It has been shown that small applied electric fields can guide the movement and migration of a variety of different cell types, thus improving *in vivo* tissue healing (Moroni et al., 2015; Ribeiro et al., 2015). Thus, conductive polymers such as polypyrrole (PPy), polyaniline (PANI), and carbon nanotubes (CNTs) incorporated into non-conductive polymers, both to provide structural support and to direct cell growth, have been tested for tissue and biomedical engineering applications (Mattioli-Belmonte et al., 2003, 2005, 2012; Harrison and Atala, 2007).

Several natural and synthetic materials could be used to generate active scaffold for tissue regeneration mainly in the form of microspheres, fibers, porous membranes, hydrogels, and sponges (Dhandayuthapani et al., 2011). Porous scaffolds have been generally obtained by traditional techniques (i.e., solvent casting/salt leaching, phase separation, gas foaming, gel casting, etc.) (Fallahiarezoudar et al., 2015) but, in order to overcome the inaccurate and limited interconnectivity pore morphology, electrospinning was also used (O’Brien, 2011). Indeed, few studies used AMT for the production of piezoelectric scaffolds (Moroni et al., 2015; Rana et al., 2015; Di Luca et al., 2016), and among these, PAM was used to realize bone-like structure scaffolds composed of CNT and polycaprolactone able to sustain osteoblast-like cell proliferation and modulate cell morphology (Mattioli-Belmonte et al., 2012).

A last generation of materials for the building up of scaffolds are polyhydroxyalkanoates (PHAs), a family of biopolyesters produced by microorganisms as intracellular carbon and energy storage compounds under unstable growth conditions (Williams and Martin, 2002; Chen, 2009). They can exist as homopolymers or copolymers of two or more hydroxyalkanoic acids, and several polymers of this family have been provided (Goonoo et al., 2016). Due to their variable composition, PHAs display diverse physicochemical properties and different rates of degradation in biological media, thereby maintaining their mechanical strength from short to prolonged amount of time (Yoshie and Inoue, 2005). Even if PHA-based scaffolds demonstrated biocompatibility with different cell types (Goonoo et al., 2016), the use of PHAs is threatened by their poor mechanical properties, as most polymers derived from natural sources. To improve physicochemical properties, thus matching biological requirements of the different human tissues, PHAs have been blended with ceramics and polymers (e.g., gelatin, silk, and collagen). These copolymers have been used mainly with traditional techniques or with electrospinning, and no data are available on the generation of composite scaffolds with other AMT (Goonoo et al., 2016). Indeed, PHA/ceramic composites showed good *in vitro* and *in vivo* bioactivity and bone regenerating potential. Moreover, the addition of angiogenic growth factors and the possible monitoring of surface/topographical properties will enable to avoid problems such as poor vascularization and cell penetration [see Goonoo et al. (2016) for details].

## Cell–Biomaterial Interaction

*In vivo*, cells are subjected to a combination of biochemical and physical factors that regulate their functional behavior (Fernandez-Yague et al., 2015).

Mechanical stimulus has been identified for a long time as a key player in the adaptation of the musculoskeletal tissues to their function, and cells are known to perceive and respond to the environmental physical cues as well as to those of tissue scaffolds. Therefore, the optimization of cell–material interactions is critical in tissue engineering, and there is increasing agreement that material physical properties (i.e., topography, geometry, porosity, and stiffness) can be used to direct guide biological results similar to the traditional approaches that involves chemistry or biomolecules (Engler et al., 2006; Mitragotri and Lahann, 2009).

Osteogenic cells respond to mechanical stimuli (Mattei et al., 2015), and several microfabricated devices have been created to induce and/or monitor cell responses to biomechanical forces and/or biochemical gradients *in vitro* (Kim and Ma, 2012). These devices can be used to analyze the effect of perfusion on human MSCs in a controlled way (Malizos and Papatheodorou, 2005; Bose et al., 2012), firmly evaluating the effect of cell seeding density and biomolecules on osteogenesis and angiogenesis (Das and Botchwey, 2011).

Different surface modification methods, such as oxidization, electrochemical deposition, or anodization *via* cathodic pre-treatment, have been used to further increase biomaterial biocompatibility and/or osteoinductivity (Hutmacher and Cool, 2007; Huang et al., 2014; Kundu et al., 2014). AMT can be used to precisely produce monotonic or graded topographical features on a biomaterial (Mattioli-Belmonte et al., 2015) and micropatterning that improves cell adhesion through focal adhesion formation (Dalby et al., 2014). The latter topological modifications can take the form of micro- and nanoscale protrusions, pits or grooves, able to direct or influence stem cell differentiation (Biggs et al., 2009; Oh et al., 2009). On the other side, since osteoclasts sense surface roughness at the resorption-sealing zone through forces applied at different heights and surface angles, biomaterials with 0.1–1 μm surface cracks can enhance osseointegration of the implant (Leijten et al., 2015).

It is clear that several factors contribute to mediating cell–material interaction, which is an intensely studied and complicated process. An ordered and regular microstructure with a smooth surface can improve material biocompatibility (Mattioli-Belmonte et al., 2008). Changing in biomaterial surface chemistry or scaffold geometry also affects cell adhesion, migration, and differentiation (Brydone et al., 2010; Bose et al., 2012).

Therefore, expressly planned micropatterning are not only capable to generate a unique topographical surface to monitor cell shape, alignment, and cell–cell and cell–matrix contact in basic stem cell biology study but could also be integrated with 3D bioprinting to develop micropattered 3D structure, thus inducing stem cell-based tissue regeneration. ECM coating on a definite topographical structure is able to induce even more precise and powerful stem cell differentiation along with soluble factors and mechanical forces (Lin et al., 2016).

## Limits in Bone Biofabrication

One of the key factors for the success of any form of transplant (either with or without the presence of scaffolds) is cell viability: transplanted cells must survive for a sufficient period in order to perform their biological function. In biofabrication approaches, the printed cells need first to survive during all processing and printing steps. Extreme environmental and culture conditions, changes before, during, and/or following printing can adversely affect cell homeostasis or result in cell damage and death (Leach and Mooney, 2004; Bose et al., 2012). Depending on the approach adopted, bioprinting can result in decreased cell viability (Leach and Mooney, 2004; Nair et al., 2009; Brydone et al., 2010; Gruene et al., 2011), with no effect documented for laser-assisted bioprinting (Schiele et al., 2010; Ali et al., 2014).

Cell viability can be also affected by the biomaterial compatibility. Many hydrogels, which are attractive given their high cytocompatibility, have been applied in tissue engineering strategies (Bryant et al., 2007; Fedorovich et al., 2011). Indeed, even if hydrogels have been planned to furnish cells with a completely hydrated 3D environment similar to the natural ECM, their poor inherent mechanical strength limits their use in 3D biofabricated bone tissue-engineered constructs (Malda et al., 2013). The increase in polymer concentration and cross-linking improves hydrogel mechanical properties, but it can affect the biofabrication process itself, both interfering with the process and/or extending the fabrication time. These aspects in turn lead to a reduction in cell viability and functionality (Hutmacher and Cool, 2007; Rouillard et al., 2011; Tang et al., 2016). In order to exceed the lack of mechanical strength of the hydrogel, hybrid 3D constructs consisting of thermoplastic biomaterials and cell-laden hydrogels have been suggested. These systems include non-woven scaffolds manufactured *via* solution electrospinning techniques and scaffolds fabricated *via* 3D printing (Visser et al., 2015).

Bone is a metabolically active tissue with an internal vasculature and osteocytes located no more than 100 μm from an intact capillary (Muschler et al., 2004). Angiogenesis, which arises spontaneously after bone grafting, is triggered by inflammation. This capillary network is transient and reverts within few weeks. The host-derived neovascularization of the implant is slow and, consequently, insufficient in the case of constructs of a relevant size. To date, vascularization remains a challenging technical obstacle in biofabrication and has prevented the development of clinically successful engineered constructs (Santo and Reis, 2010; Nguyen and Burg, 2015). In order to solve this issue, strategies involving the use of coculture systems, perfusion bioreactors, biomaterials, and growth factors to direct cell behavior are under investigation (Allori et al., 2008; Kyriakidou et al., 2008; Nguyen et al., 2012; Mercado-Pagan et al., 2015; Nguyen and Burg, 2015). Microscale technologies provide plasticity in generating accurate 3D architectures with embedded vascularized and capillary networks. Present methods include the formation of a ditch precasted into one layer before a second layer is aligned and deposited, forming laminated channel(s) or grooves in a repetitive way (Miller et al., 2012; Costa et al., 2014). Using this method, Moroni et al. (2006) formed microscale 3D scaffolds with organized hollow fibers with governable diameter and thickness that could be used as a vascularized network.

Scaffold pores are essential for the formation of bone tissue, since they enable cell migration and ingrowth as well as nutrient diffusion for cell survival (Karageorgiou and Kaplan, 2005). In general, scaffolds with pore sizes greater than 50 μm allow nutrient diffusion but show lower cell adhesion and intracellular signaling. For an effective cell growth, pore size must be “tailored” based on different cell types needs. For instance, larger pores are useful for osteoblasts growth, while fibroblasts preferred smaller pores (Oh et al., 2007). Narayan and Venkatraman (2008) reported that the *in vitro* growth of endothelial cell on PLGA scaffolds was enhanced on 5–20 μm pore sizes, with lower interpore distance. However, this was in contrast to some *in vivo* studies that showed as a higher porosity permits for faster bone ingrowth and vascularization. Improved bone formation was observed in hydroxyapatite scaffolds with 300–400 μm pore sizes implanted in rats, suggesting that a fast scaffold vascularization determines an osteogenic microenvironment (Klenke et al., 2008; Bai et al., 2010). Conflicting results in these studies stress the limitations of *in vitro* researches in predicting *in vivo* results as well as the requirement to assess the best pore sizes for each cell type used for bone tissue engineering. Moreover, porous or rough materials integrate in a less fibrotic, better-vascularized way in comparison with smooth, compact forms.

An important reason in implant failure is associated with a foreign body reaction: biocompatible biomaterials become encapsulated and are phagocytized by macrophages. Indeed, it has also been shown that porous biomaterials with pore of 30–40 μm in size displayed excellent healing with a pro-healing functionality of macrophages, regardless of polymer composition or implant site (Osathanon et al., 2008).

## Clinical Translation

A basic problem for a positive translation of any cell therapy for regenerative medicine purposes is still their large-scale production. In this respect, the bioprocessing phases of the producing process must be reproducible and scalable, in accordance with good clinical and manufacturing practice standards, safe for patients, and economically sustainable (Martin et al., 2014). Regardless of high resolution and reproducibility, laser-assisted bioprinting techniques offer low number and small-scale manufacture of constructs (Guillotin et al., 2014), and inkjet bioprinting has similar limitations (Guillotin and Guillemot, 2011), while extrusion methods shows a higher resolution than inkjet-based printing in producing structures suitable for clinical use (Khatiwala et al., 2012).

The standard file format used to control AMT bioprinters is STL. While it works for solid objects with small complexity, STL is an unfeasible format if internal pore architecture is an integral part of the computer-aided design (Melchels et al., 2012). New methods to create porous models from medical imaging-derived data are therefore under evaluation in order to enable the study of the effects of biomechanical forces on bone remodeling (Wang and Mondry, 2005).

*In vivo* animal models can produce several relevant data on bone repair processes and testing the effectiveness of bioprinted or biofabricated bone constructs. These researches generally encompass big numbers of animals that raise ethical concerns and are expensive. The development of *ex vivo* organ cultures (Smith et al., 2014) as well as the use of chick chorioallantoic membranes for the study of vascularization, biomaterial compatibility, and growth factors (Nowak-Sliwinska et al., 2014) have facilitated the reduction of animals used for the study of bone repair process. Despite these advances, there are still few comparable data in the literature on long-term *in vitro* and *in vivo* characterization of bioprinted and biofabricated bone constructs. The improve accuracy of *in silico* predictive models would also reduce the numbers of animals used in *in vivo* studies (Fedorovich et al., 2011; Ferretti et al., 2015; Vozzi et al., 2016).

Modern microscopy techniques have helped in the evolution of biomaterials, from their composition to their interactions (Vielreicher et al., 2013). A major advantage of these techniques in the analysis of tissue constructs is that most of them are non-destructive. Table 7 summarized some of the microscopic techniques used to study bone tissue engineering.

**Table 7:** **Imaging methods used in bone tissue engineering**.^a^

Method	Physical principles	Imaging depth	3D imaging	Acquisition speed	Invasiveness	Specificity
μCT	X-ray diffraction	Whole body	Excellent	Average	Low for hard tissue	Average
Confocal light microscopy	1-photon fluorescence: 200	<100 μm	Excellent	Good	Low	Excellent
Light microscopy	Light diffraction and interference	N.A.	Poor	Excellent	Low	Low without staining
SEM/TEM	Electron diffraction	N.A./<200 nm	Very poor	Very poor	Very high	Excellent
Non-linear optical microscopy	2-photon fluorescence/second harmonic generation/coherent anti-Stokes Raman scattering	<1,000 μm	Excellent	Good	High	Excellent

*μCT, microcomputed tomography; SEM, scanning electron microscopy; TEM, transmission electron microscopy; N.A., not applicable; 3D, three dimensional*.

*Invasiveness indicates the degree of tissue damage*.

*^a^Modified from Tang et al. (2016) and Vielreicher et al. (2013)*.

When it is necessary for imaging cells within scaffolds, the limited penetration depths for most microscopy techniques is a major limitation. The introduction of multi-photon microscopy as well as advances in microcomputed tomography has allowed for slice-wise optical sectioning and 3D reconstruction of these constructs. These improvements overcome some of the limitations associated with traditional light microscopy. However, the computing power and storage space necessary to perform, analyze, and collect the data obtained with these methods is massive, even by today’s standards in information technology.

Finally, biofabrication is a quickly developing field with the capacity to change drastically the actual medical treatments. However, this idea requires integrated bioprinting platforms able to manage multiple materials to tissue constructs with structural integrity and of clinically relevant scales. Recently, Kang et al. (2016) developed such a platform and effectively produced different tissue types *in vivo*, opening a breakthrough for the clinical translation of this technology, even if to achieve this final goal, further steps and challenges remain (Malda and Groll, 2016).

## Conclusion

Biofabrication using AMT offers a defined and organized approach for bone tissue generation in comparison with traditional techniques. Nevertheless, there are still significant challenges with biofabrication for the development of clinically relevant bone constructs. These problems are not only relative to the existing limitations in AMT but are also due to the possibility to obtain an appropriate vascularization of the structures as well as correct spatiotemporal biochemical and mechanical stimuli, to maximize osteogenesis and osseointegration. Advances in AMT, computational modeling, medical imaging and microscopy technology, bioreactor design, and biomaterial and drug development are therefore mandatory in order to face difficulties and improve the cost-effectiveness of biofabricated bone for clinical therapy, which at present is poor. Moreover, the correct cell selection for an effective clinical result must be taken into consideration.

It must also be underlined that all improvement in bone biofabrication will surely aid the knowledge and understanding of skeletal stem cell biology, cell interactions, and responses to external stimuli for bone development, formation, and remodeling related to the aging of population.

## Author Contributions

MB conceived and critically revised the whole manuscript. MO drafted and revised the section relative to cell sources and biomolecules. MF drafted and revised the section relative to the limits of biofabrication techniques. RP critically revised the whole manuscript.

## Conflict of Interest Statement

The authors declare that the research was conducted in the absence of any commercial or financial relationships that could be construed as a potential conflict of interest.

## Abbreviations

ALP, alkaline phosphatase; AMTs, additive manufacturing technologies; ASCs, adipose tissue-derived stem cells; BM-MSCs, bone marrow stromal cells; BMP, bone morphogenetic protein; CAD, computer-assisted design; CNTs, carbon nanotubes; CT, X-ray computed tomography; ECM, extracellular matrix; EPC, endothelial progenitor cells; FGF, fibroblast growth factor; HUVEC, human umbilical vein cells; IGF, insulin-like growth factor; MAPK, mitogen-activated protein kinase; MRI, magnetic resonance imaging; MSCs, mesenchymal stromal cells; PAM, pressure-activated microsyringe; PCL, polycaprolactone; PDGF, platelet-derived growth factor; PANI, polyaniline; PEGDMA, poly(ethylene glycol) dimethylacrylate; PEG-PLGA, poly (ethylglycol)-poly (lactic-co-glycolic acid); PHAs, polyhydroxyalkanoates; PLGA, poly (lactic-co-glycolic acid); μCT, microcomputed tomography; PPy, polypyrrole; PTH, parathyroid hormone; PTHrH, parathyroid hormone-related protein; SEM, scanning electron microscopy; S-MSCs, skin-derived multipotent stromal cells; SPECT, single-photon gamma rays; STL, Standard Tessellation Language; 3D, three dimensional; TEM, transmission electron microscopy; TGF, transforming growth factor; VEGF, vascular endothelial growth factor.
